# Function and Molecular Mechanism of the DNA Damage Response in Immunity and Cancer Immunotherapy

**DOI:** 10.3389/fimmu.2021.797880

**Published:** 2021-12-14

**Authors:** Zu Ye, Yin Shi, Susan P. Lees-Miller, John A. Tainer

**Affiliations:** ^1^ Department of Molecular and Cellular Oncology, and Department of Cancer Biology, The University of Texas MD Anderson Cancer Center, Houston, TX, United States; ^2^ Department of Immunology, Zhejiang University School of Medicine, Hangzhou, China; ^3^ Division of Pediatrics, The University of Texas MD Anderson Cancer Center, Houston, TX, United States; ^4^ Department of Biochemistry and Molecular Biology, Robson DNA Science Centre, Charbonneau Cancer Institute, University of Calgary, Calgary, AB, Canada

**Keywords:** DNA repair, immune response, DNA damage, cGAS-STING, innate immunity, adaptive immunity, immunomodulatory, cancer therapy

## Abstract

The DNA damage response (DDR) is an organized network of multiple interwoven components evolved to repair damaged DNA and maintain genome fidelity. Conceptually the DDR includes damage sensors, transducer kinases, and effectors to maintain genomic stability and accurate transmission of genetic information. We have recently gained a substantially improved molecular and mechanistic understanding of how DDR components are interconnected to inflammatory and immune responses to stress. DDR shapes both innate and adaptive immune pathways: (i) in the context of innate immunity, DDR components mainly enhance cytosolic DNA sensing and its downstream STimulator of INterferon Genes (STING)-dependent signaling; (ii) in the context of adaptive immunity, the DDR is needed for the assembly and diversification of antigen receptor genes that is requisite for T and B lymphocyte development. Imbalances between DNA damage and repair impair tissue homeostasis and lead to replication and transcription stress, mutation accumulation, and even cell death. These impacts from DDR defects can then drive tumorigenesis, secretion of inflammatory cytokines, and aberrant immune responses. Yet, DDR deficiency or inhibition can also directly enhance innate immune responses. Furthermore, DDR defects plus the higher mutation load in tumor cells synergistically produce primarily tumor-specific neoantigens, which are powerfully targeted in cancer immunotherapy by employing immune checkpoint inhibitors to amplify immune responses. Thus, elucidating DDR-immune response interplay may provide critical connections for harnessing immunomodulatory effects plus targeted inhibition to improve efficacy of radiation and chemotherapies, of immune checkpoint blockade, and of combined therapeutic strategies.

## Introduction

Key cancer hallmarks critically include genomic instability, immune modulation, and altered DNA damage and other stress responses to favor overall cell survival (1, 2). Every day, tens of thousands of damaged DNA lesions occur in each human cell that could impact cell survival and genomic integrity (3). Importantly, the outcome of this DNA damage depends directly upon the nature and actions of the DNA damage response (DDR). Lesions become accurately or inaccurately repaired or left as unrepaired mutations depending upon the DDR. As a result, evolutionary selection ensures that the DDR is a carefully orchestrated response system consisting of multiple signaling pathways that largely maintain genomic stability and fidelity despite high levels of DNA damage (4, 5). Yet, comprehensive analyses of cancer genome databases reveal non-B DNA, mitochondrial dysfunction, and the activation of DNA repair/cell cycle pathways as major factors driving somatic mutation loads in cancer cells (2, 6). From a mechanistic standpoint, the positive correlations of these factors with mutations in cancer cells likely arise from increased reactive oxygen species (ROS), oncogenic replication and transcription stress, and the combination of resulting excessive DNA damage plus its escape from accurate repair.

In particular, DDR are activated by replication obstacles in proliferating cells that lead to replication stress: replication fork stalling, collapse or breakage, such as lesions from oxidation, deamination and alkylation, DNA breaks, protein-DNA cross-links, and non-B DNA structures including R-loops (RNA-DNA hybrids formed by replication-transcription conflicts) (7–9). DNA damage and activation of the DDR from endogenous replication stress are seen at pre- or early stages of oncogenesis, and adaptation to replication stress acts in tumor development (10). In breast-cancer susceptibility gene 2 (BRCA2)-deficient cancer cells, the inactivation of replicative stress response factors (e.g. poly (ADP-ribose) polymerase [PARP1] or ATM and Rad3-related [ATR] inhibition) triggers cyclic GMP-AMP synthase (cGAS)-STING-mediated innate immune responses (11, 12). Furthermore, inherent DNA repair defects in tumors may develop mutation-driven neoantigens that can cause the immune system to recognize the tumor cells as foreign while also increasing the amount of cytosolic DNA to trigger a cGAS-STING response. Thus, the DDR that largely protects against DNA damage in normal cells can often be defective or defeated in proliferating cancer cells with consequent impacts on immune responses. This finding implies a fundamental importance of DDR for cancer biology, for the elucidation of cancer vulnerabilities, and for optimal applications of immunotherapy.

The DDR machinery can conceptually be divided into at least six distinct DNA repair pathways responding to different types of DNA damage: (i) homologous recombination (HR), which repairs double-strand breaks (DSBs) using a homologous DNA template; (ii) non-homologous end joining (NHEJ), which repairs DSBs without a corresponding template; (iii) alternative end-joining (A-EJ), which repairs DSBs with insertion and deletion errors by employing micro-homology; (iv) nucleotide excision repair (NER), which repairs bulky DNA lesions globally or coupled to transcription; (v) mismatch repair (MMR), which repairs DNA single-strand breaks (SSBs) predominantly generated during DNA replication and recombination processes plus mismatches that escaped replication fidelity; and (vi) base excision repair (BER), which removes bases damaged by oxidation, alkylation, deamination, and methylation to avoid replication and transcription blocks and errors (4, 13–15).

The various DDR pathways share similarities in how they respond to the stress of damaged DNA, whereby a damage sensor that can also be a repair effector [e.g., RPA, MUTY, PARP1, Ku70/80, MRE11-RAD50-NBS1 (MRN) complex] recognizes specific DNA damage types (single-stranded DNA, base mismatches, SSBs, and DSBs) before recruiting and activating downstream transducer kinases (such as ATM, ATR, DNA-PKcs), which in turn transduce the signal to effector proteins (such as MRN, CHK1, EXO5, p53, RAD51, and BRCA1/2). The ensuing complexes ultimately orchestrate repair by employing damage removal and sequence replacement by handoffs or dynamic machinery that have evolved to avoid the release of toxic and mutagenic DNA intermediates (15, 16). Thus, the DDR is an ancient and evolutionarily conserved mechanism that is essential for genome stability and cell survival (17, 18).

As the major stress response system essential for surviving infection, the immune response is an evolved network of proteins and complexes that respond to invading pathogens and their associated toxins. Importantly, DDR defects can lead to imbalance between DNA damage and repair, impairing tissue homeostasis and leading to replication and transcription stress, mutation accumulation or outright cell death: this imbalance can drive tumorigenesis as well as secretion of inflammatory cytokines, and aberrant immune responses (19–23). All organisms possess mechanisms to detect and eliminate foreign pathogens *via* the innate immune system. Additionally, higher vertebrates employ a sophisticated adaptive immune system that includes antibodies as well as B and T lymphocytes with virtually limitless repertoires of receptors that mediate neutralization of foreign pathogens and removal malignant cells (24–26). To stimulate strong anti-tumor immune responses, cancer immunotherapy typically employs immune checkpoint inhibitors for the PD-1/PD-L1 and CTLA-4 pathways to amplify immune system responses and also to harnesses responses to neoantigens that are primarily tumor-specific antigens resulting from the higher mutation load in tumor cells (27–29). The validity of the PD-1/PD-L1 approach requires the functional MHC class I complex, which itself is often deleted during tumor evolution to escape immune regulation (30).

For clarity this review is divided into five overall sections: 1) Introduction, 2) DDR in innate immunity, 3) DDR in adaptive immunity, 4) DDR inhibition in antitumor immunity, and 5) Summary and prospects. Within these sections and their subsections, we furthermore consider how these critical and seemingly distinct DDR and immune stress responses are intertwined and where defining their interconnections may enable novel insights into etiology and advanced molecular-based treatment of cancer and other human diseases.

## DDR in Innate Immunity

Innate immunity is the first immunological defense system against pathogens. Activation of the innate immune response relies on Pattern Recognition Receptors (PRRs). These PRRs detect Damage-Associated Molecular Patterns (DAMPs) or Pathogen-Associated Molecular Patterns (PAMPs) to initiate a signaling cascade resulting in production of interferons (IFNs), cytokines and chemokines (24, 26, 31). Importantly, non-self nucleic acids are the most well-characterized stimuli for the innate immune response (32, 33); furthermore, endogenous cytosolic DNA released from the nucleus or mitochondria stimulates the innate immune system.

DNA damage caused by genotoxic stresses or DNA damage stimulus (e.g., cytotoxic chemotherapy and radiation) can create cytosolic chromosomal fragments that may be recognized by cGAS, a cytosolic DNA sensor. Cytosolic exposure of chromosomal DNA by micronuclei rupture, breakage of chromatin bridges, or disintegration of micronuclei-like cytosolic chromatin fragments activates cGAS (34). Once bound to cytosolic DNA, activated cGAS can form dimers and multimer assemblies that undergo liquid–liquid phase separation to form biomolecular condensates that amplify cGAS activation (35). Activated cGAS produces 2´-3´cGAMP (cGAMP) as a second messenger to function in both the host cell and adjacent cells *via* secretion or by passage through gap junctions, which contributes to the bystander response to radiotherapy in non-irradiated neighboring cells (36–40). In the presence of cGAMP, STING is relocated from the ER to Golgi, where it recruits and activates TANK-binding kinase (TBK1), that activates interferon regulatory factor 3 (IRF3) and NF-κB signaling (41). Activated IRF3 and NF-κB then induce transcription of innate immune response genes, including IFNs and cytokines (36, 37, 42).

Interestingly, cGAS is also found in the nucleus. Nuclear cGAS is inactivated by its acidic patch binding to nucleosome core particles, which prevents DNA binding, thus preventing autoreactivity (34). Moreover, nuclear cGAS is recruited to DNA damage sites by γH2AX, which promotes its interaction with Poly (ADP-ribose) polymerase 1 (PARP1) and impedes formation of PARP1-Timeless complex to thereby suppress HR but not NHEJ (43, 44).

Another important cytosolic DNA sensor is γ-interferon-inducible protein-16 (IFI16). Like cGAS, IFI16 can detect both self and non-self dsDNA to promote IRF3 and NF-κB -dependent interferon production *via* STING (26, 45).

**Figure 1:**
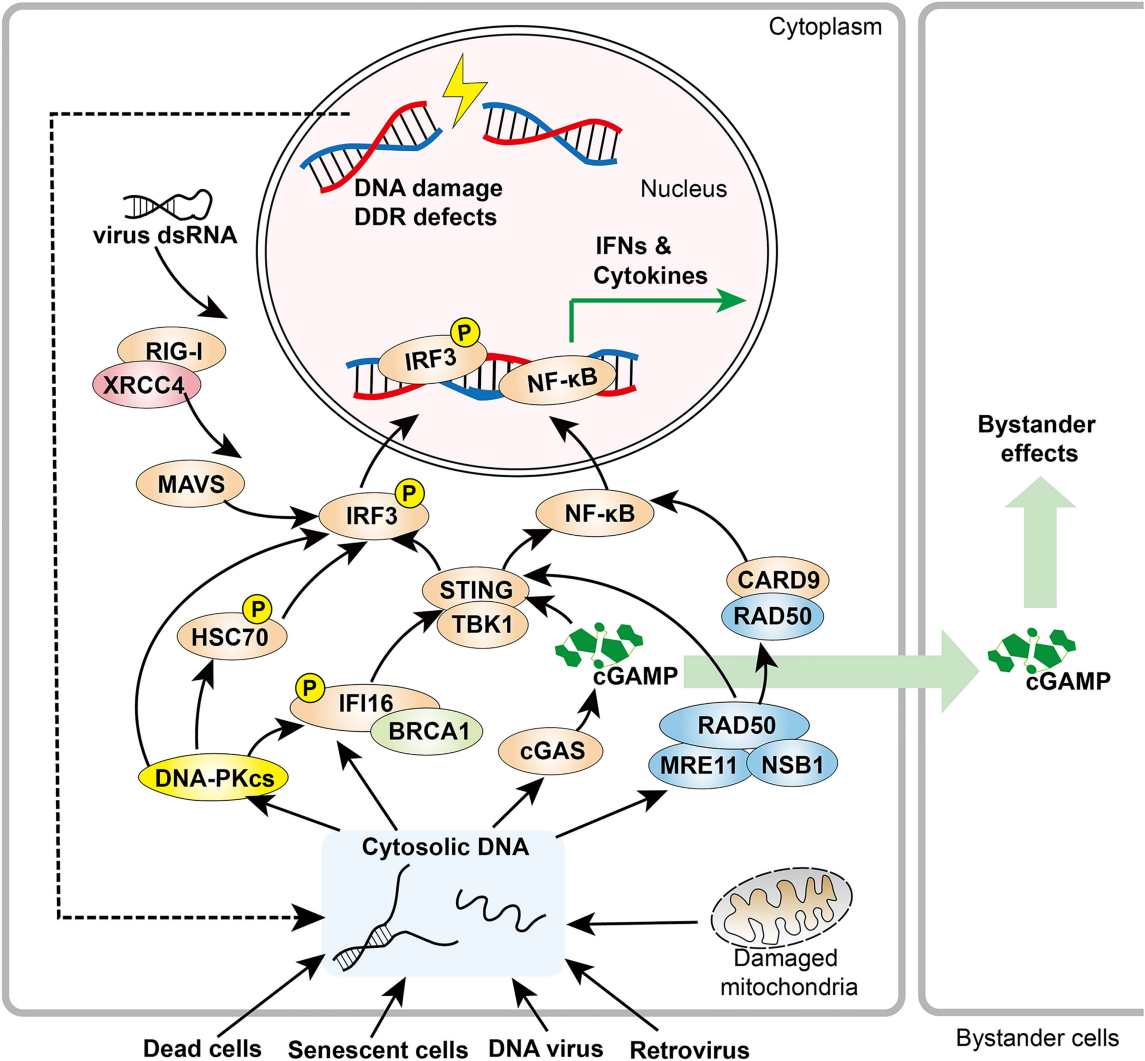
Overview of DDR components in innate immune responses. DDR factors, including DNA-PK and MRE11, promote cytosolic DNA sensing signaling pathways. When activated by cytosolic DNA, cGAS produces cGAMP, a soluble second messenger that initiates STING-IRF3 signaling both within the host cell and adjacent cells. In addition, RAD50 associates with CARD9, leading to NF-κB activation and downstream cytokine production. XRCC4 interacts with RIG-I, which promotes the RIG-I-MAVS-IRF3 pathway.

Emerging data reveal that DNA repair pathways and cytosolic pathological DNA sensing pathways have overlapping effectors that recognize and respond to damaged nuclear DNA, cytosolic endogenous DNA, or foreign DNA (46). These observations provide compelling evidence for inextricable links between the DDR and innate immune responses (**Figure 1**).

### DDR Deficiency or Inhibition Enhances Innate Immune Responses

Interference in DDR signaling elicits innate immune responses. One of the most well-studied examples is PARP inhibition. PARP inhibition generates cytosolic chromatin fragments and significantly potentiates cGAS-STING-dependent immune responses (11, 47–54). Similarly, DNA damage as a result of cytotoxic chemotherapy, ionizing radiation (IR), metabolism, and deficiency of other DDR elements (including BRCA2, ATM, CHK1, RPA, RAD51, TREX1 and FANCD2), also leads to increased IFN signaling–mediated immune responses (11, 19, 55–61).

The RecQ–like BLM helicase partners with EXO5 and EEPD1 nucleases for stalled DNA replication restart and maintenance of genome integrity (62, 63). BLM deficiency in Bloom syndrome (BS) causes increased expression of inflammatory genes through the cGAS–STING–IRF3 pathway, suggesting it prevents unchecked inflammatory gene responses (64). ROS from radiation therapy or cell stress lead to cGAS-STING-mediated immune responses to cancer from DSBs as well as oxidative adducts that must be removed by DNA glycosylases, such as endonuclease VIII (Nei)-like proteins (NEIL) and oxoguanine DNA glycosylase (OGG1) (65–67). Furthermore, high levels of ROS that are not efficiently reduced by superoxide dismutases and catalase can leave unrepaired 8-hydroxyguanosine (8-OHG) (68, 69). 8-OHG stabilizes DNA against degradation by the cytosolic DNA exonuclease TREX1, leading to accumulated cytosolic DNA and increased cGAS activation (70). This ROS effect can be amplified by vicious cycles of oxidative damage and iron release from ROS-sensitive 4Fe-4S co-factors in multiple replication and repair proteins (62, 71–73).

Metabolism and innate immunity converge at the mitochondria, which can orchestrate innate immune signaling pathways in different cancer-relevant metabolic scenarios including a link to PARylation and cell death (74, 75). Metabolic cues including nucleotide imbalance can stimulate the release of mtDNA from mitochondria that drives an interferon response with MRE11 playing a leading role (76). The fundamental importance of DNA breaks in promoting such immune responses is evidenced by the observation that mtDNA breaks synergize with nuclear DNA damage to mount a robust cellular immune response (77).

In general, unresolved DNA damage can act as a mediator linking the DDR and immune recognition, and this can involve the formation of micronuclei as an initiating event in a cascade promoting genomic instability and innate immune responses (78, 79). Moreover, genome instability and imperfect cell cycle checkpoints in tumor cells enhance formation of micronuclei, making them more susceptible to targeting of the innate immune response (5, 22, 79). DNA damage responses occur in minutes to hours. Yet, there is a delayed onset of days for inflammatory cytokines that modify tumor microenvironment by immune cell recruitment as critical for local and systemic (abscopal) tumor responses to radiotherapy.

### DNA-PK in Innate Immune Response

DNA-dependent protein kinase (DNA-PK) is a trimeric nuclear complex that functions as a central integrator of the DSB repair system. The protein complex consists of a large catalytic subunit, DNA-PKcs, and the Ku70/80 heterodimer (Ku70/80) which recognizes DSB ends (80). DNA-PKcs is a Ser/Thr protein kinase and the largest member of the phosphatidylinositol 3-kinase (PI3K)-related kinase (PIKK) family (81). Once activated by Ku70/80, DNA-PK undergoes autophosphorylation and is then positioned to phosphorylate other repair effectors and promote a synaptic complex for ligation of two dsDNA ends (80, 82–86). In recent decades, emerging evidence revealed that DNA-PK is a critical component of innate immunity against multiple viruses, including human immunodeficiency virus (HIV), Herpes Simplex Virus 1 (HSV-1), alphavirus M1, and vaccinia virus (87–92). As such, DNA-PK is a key DNA sensor that modulates innate immunity through several critical components of innate immune pathways.

In STING-dependent DNA sensing pathways, cGAS, IFI16, and IRF3 are substrates for DNA-PK (89, 93, 94). However, the role of DNA-PK within the cGAS-STING pathway remains controversial. One recent study reported that DNA-PK directly phosphorylates cGAS to suppress its enzymatic activity and thus attenuate innate immune responses (93). To this end, DNA-PKcs deficiency caused by missense mutations in its coding gene, *PRKDC*, leads to an increased inflammatory response in both human and mouse cells (93). In contrast, a pioneering study showed that DNA-PK interacts with and phosphorylates IRF-3, thus promoting its nuclear translocation (94). In a systematic profiling study, DNA-PKcs directly phosphorylated the DNA sensor IFI16 and promoted IFI16-driven cytokine responses (89). Furthermore, regardless of its partner cGAS, STING can localize to the inner nuclear membrane in breast cancer tumor samples and promote cancer cell survival by resistance to DNA-damaging agents through interacting with DNA-PK (95). Therefore, further studies are warranted to better understand mechanisms governing DNA-PK substrate selection within the context of the innate immune response.

As described above, although a potentially suppressive role of DNA-PK on cGAS was reported which may be context dependent, most studies suggest that DNA-PK promotes a STING-dependent innate immune response (96–100). Mechanistically, the HEXIM1-DNA-PK-paraspeckle components-ribonucleoprotein complex (HDP-RNP), containing DNA-PK subunits and paraspeckle proteins, is required for foreign DNA sensing through the cGAS-STING pathway. The HDP-RNP interacts with cGAS, and when stimulated by cytosolic DNA, the paraspeckle proteins from the complex are released to recruit STING and activate DNA-PK and IRF-3. Knockdown of HDP-RNP subunits including Ku70, the DNA binding subunit in DNA-PK, resulted in loss of IFN stimulatory DNA–mediated immune response (97). In addition, Ku70 was identified as a cytosolic DNA sensor that translocates to the cytoplasm to form a complex with STING and induce production of IFN-λ1 (98, 99).

Besides STING-dependent DNA sensing mechanisms, DNA-PK also acts as a DNA sensor to trigger a robust and broad antiviral response in a STING-independent DNA sensing pathway (SIDSP) in human cells, but not in laboratory mice (101), perhaps a reflection that DNA-PK levels in human cells are much higher than in mouse cells (102–104). A recently characterized DNA-PK partner is LINP1, a lncRNA that can recruit multiple DNA-PK assemblies and promote formation of phase condensates (105). As LINP1 is present in both cytoplasm and nucleus, it will be important to test its potential role in cytosolic immune activation.

Overall, DNA-PK is considered a cytosolic DNA sensor for both STING-dependent and -independent DNA sensing pathways. The extent to which the role of DNA-PKcs in the innate immune response is distinct from its well-characterized nuclear functions in NHEJ is under active investigation.

### MRN Complex in Innate Immunity

MRN, a core orchestrator that senses DSB damage and activates DNA repair cascades, is required to maintain genome integrity (13). In recent years, the MRN complex, which acts in DSB sensing, stabilization, signaling, and effector scaffolding (106), has furthermore been found to localize to viral replication sites and trigger innate immune responses (107–109).

An exemplary MRN role in regulating innate immunity comes from the meiotic recombination 11 homolog A (MRE11) nuclease subunit, which recognizes and processes DSB DNA ends as a part of HR repair, replication fork processing, and telomere length maintenance (110, 111). MRE11 has both endonuclease and exonuclease activities that, together, initiate HR repair (112). Furthermore, MRE11 functions as a key cytosolic DNA sensor in recognition of a broad spectrum of dsDNA and activates STING trafficking and type I IFN production in various cell types (108).

An intriguing observation is that nuclease activity is not required for the cytosolic DNA-sensing function of MRE11, which reinforces the notion that besides their nucleotide processing activity DDR nucleases also function to recognize and sculpt specific DNA structures (113–116). In fact, the nuclease-inactive mutant form of MRE11 triggers an even higher immune response than the wild-type form. Therefore, MRE11 may act as a regulatory switch within the STING-dependent immune response, initially functioning as a DNA sensor to activate STING-mediated signaling, then subsequently working as a nuclease to suppress excessive immune responses (108). Obviously, further studies are required to better elucidate the pro- and anti-immune–modulating mechanisms of MRE11 in STING-dependent signaling. Nevertheless, these data suggest that STING-mediated signaling may be activated by one of the existing MRE11 inhibitors (112, 117). It will also be interesting to see if the adaptor regulator GRB2 complex with MRE11, which promotes HR and suppresses A-EJ in the nucleus, plays a role in STING-mediated signaling (118). Intriguingly, multiple GRB2 molecules can also bind to Linker of Activation of T cells (LAT) to mediate its oligomerization, which is important for T-cell signaling under limiting stimulating conditions. Furthermore, GRB2 promotes metabolic reprogramming to support T cell activation (119–121). These and other data support the notion that tight protein and DNA binding plus conformational sculpting can regulate activities and switch DNA repair pathways (122).

MRE11 mutations that result in loss of binding ability to Nijmegen breakage syndrome protein 1 (NBS1) induce type I IFN comparable to wild-type MRE11 (108). This finding suggests that NBS1 is not instrumental for sensing cytosolic DNA and provoking an immune response. This concept is consistent with the mechanistic role implied by the NBS1 structure and its MRE11 interface, to flexibly restrict DNA end processing and homologous recombination activities to the vicinity of DSBs (123). On the basis of prior data showing that NBS1 loss promotes cytosolic MRE11 distribution (124), we propose that a deficiency of NBS1 may enhance cytosolic DNA sensing by MRE11.

The third component of the MRN complex is the ATP-binding cassette-ATPase (RAD50). MRE11 nuclease activity is regulated by ATP-dependent RAD50 helical coiled-coil conformations that switch the MRE11-RAD50 complex between DNA tethering, ATM signaling, and strand resection (125, 126). RAD50 plays an important role in innate immunity *via* a STING-independent signaling pathway (109). RAD50 binds a proinflammatory signaling adaptor amino-terminal caspase-recruitment domain (CARD9) through its structurally defined zinc-hook region (127). Together with MRE11, RAD50 recognizes cytosolic DNA and interacts with CARD9, which leads to the recruitment of Bcl-10 to induce NF-κB activation and pro-inflammatory cytokine IL-1β generation (109).

### Other DDR Factors in Innate Immunity

BRCA1, which together with the MRN complex plays a central role in HR DNA repair, interacts with IFI16 (128, 129). In herpesvirus-infected cells, BRCA1 is required for IFI16-mediated recognition of foreign DNAs, association with STING, and subsequent IFN-β production (128). Aside from DNA virus sensing, X-ray repair cross-complementing group 4 (XRCC4), a DNA ligase IV (LIG4)-associated protein essential for NHEJ (130–132), acts in an RNA-sensing pathway through interaction with retinoic acid-inducible gene I (RIG-I) (133). XRCC4 promotes oligomerization and ubiquitination of RIG-I, which results in enhancement of the RIG-I-MAVS-IRF3-type I IFN signaling cascade and subsequent suppression of RNA virus replication in host cells. Reciprocally, RIG-I competes with LIG4 to interact with XRCC4, and therefore it impedes XRCC4-dependent NHEJ cascades and hinders retrovirus integration into the host genome by suppressing the NHEJ pathway (133). This finding highlights the critical role of XRCC4 in defense against RNA viruses and in potentiating innate immune response.

## DDR in Adaptive Immunity

Unlike the innate immune system, characterized by rapid sensing and elimination of pathogens as first line of defense, the adaptive immune system provides broader and more accurate discrimination between self and non-self immunogens based on the process of positive and negative selection during lymphocyte development (25). A robust adaptive immune response to any pathogen or biological macromolecule seen for the first time takes weeks to mount. However, subsequent exposure to the same pathogen promotes a rapid “memory” response that is often magnitudes stronger than the response following the first exposure. In adaptive immunity, the DDR is essential for lymphocyte development by facilitating the assembly and diversification of antigen receptor genes (134, 135). Thus, DDR deficiencies are linked with immunological disorders, including autoimmune diseases, such as systemic sclerosis, pediatric systemic lupus erythematosus, and severe sepsis (136–139).

Ataxia-telangiectasia (A-T), a disorder arising from *ATM* germline mutations, was one of the first-identified disorders whereby immunodeficiency was associated with an aberrant DDR (140–142). Missense mutations of *PRKDC*, which encodes the catalytic subunit in DNA-PK, were also found in patients with the organ-specific autoimmunity phenotype (93, 143). In addition, autoantibodies directed against Ku 70/80 were detected in autoimmune patient sera (144, 145). Indeed, Ku was first identified *via* autoantibodies in sera from patients with the autoimmune disease polymyositis-scleroderma overlap syndrome (146).

**Figure 2:**
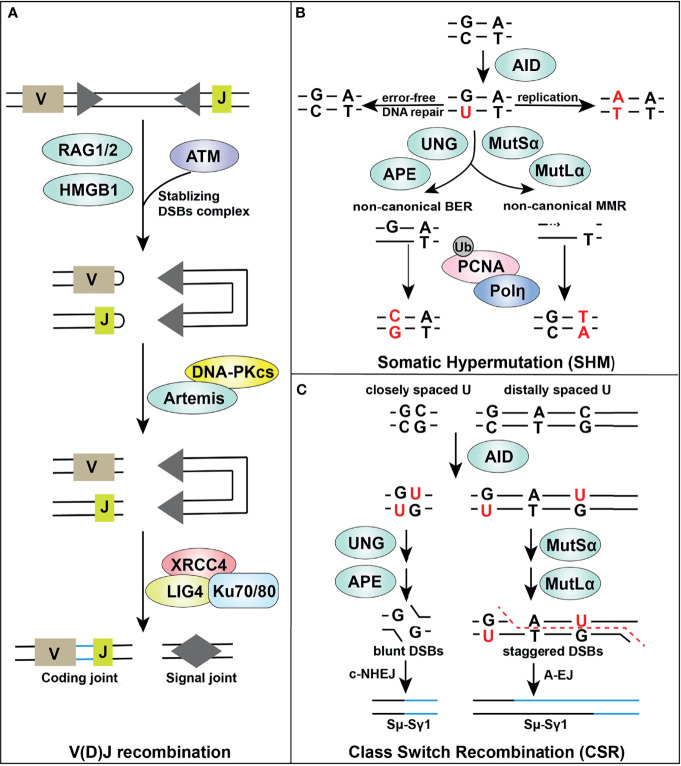
Overview of DDR components in adaptive immune responses. Certain DDR signaling pathways, such as MMR, BER, NHEJ, and A-EJ, are required in V(D)J recombination **(A)**, SHM **(B)** and CSR **(C)** processes, supporting successful lymphocyte development.

Adaptive maturation of T and B lymphocytes is guided by the “blueprint” of different cell surface receptors. During the process of lymphocyte maturation, three highly regulated processes, including variable, diversity, and joining [V(D)J] recombination, class-switch recombination (CSR), and somatic hypermutation (SHM) together with negative and positive selection (147), lead to generation of a functional, genetically diverse, and non-autoreactive antigen receptor repertoire. Interestingly, these processes naturally generate DSBs and/or trigger a DDR in adaptive immunity (**Figure 2**) (136, 148). In this section, we review these pathways highlighting roles of important DNA repair factors.

### DDR in V(D)J Recombination

V(D)J recombination occurs in G1 phase of naive, progenitor T and B lymphocytes, and enables rearrangement of gene segments at both immunoglobulin and T-cell receptor loci in a lineage specific and developmental stage specific manner (148, 149). V(D)J recombination is initiated by the recombinase activating gene (RAG) endonucleases RAG1 and RAG2, which is directed by RAG recognition sequences (recombination signal sequences [RSS]) (**Figure 2A**). The RAG complex creates a nick between the coding segment and the flanking RSS which leads to a DNA hairpin at the ends of the gene segment containing the coding regions (coding-ends) and a blunt-ended DSB at the end of the RSS, so called signal-ends. Alignment of coding regions, excision, and formation of hairpin-ended coding-ends and blunt-ended signal-ends takes place within the RAG1/2 complex, aided by HMGB1 (148, 149). RAG-mediated DSBs are processed by the NHEJ machinery to assemble genes encoding immunoglobulin, and heterodimeric B- and T-cell receptors (150–152). The rapid repair of RAG-mediated DSBs by NHEJ is essential for normal lymphocyte development. Failure to repair RAG-mediated DSBs in immature B cells leads to a DDR including ATM-mediated upregulation of NF-κB signaling (134, 153–157).

DNA-PKcs in complex with Artemis, a member of the metallo-β-lactamase protein family, is required for successful V(D)J recombination and lymphocyte development. DNA-PKcs interaction is required for Artemis endonuclease and exonuclease activities for the RAG-mediated hairpin-opening step in V(D)J recombination and for 5’ and 3’ overhang processing in NHEJ (158). The two coding-ends, each terminating with a DNA hairpin, are released from the RAG1/2 complex first. Prior to rejoining, the DNA hairpins are opened by the Artemis-DNA-PKcs complex, which cleaves 3’ to the apex of the DNA hairpin. Artemis requires DNA-PKcs for its hairpin opening activity but how this occurs is still an open question (159, 160). Nevertheless, both the interaction of DNA-PKcs with Artemis, and DNA-PKcs phosphorylation are important for Artemis activation (161, 162).

Irrespective of the mechanism, DNA-PKcs protein and Artemis are both required for opening the coding-end hairpins, as the unopened hairpins accumulate in cells lacking either Artemis or DNA-PKcs (158). Indeed, mice, dogs and horses with mutations that compromise DNA-PKcs protein levels are characterized by radiation sensitivity (due to defects in NHEJ and DSB repair) as well as severe loss of T and B cells resulting in severe combined immunodeficiency (SCID) (163–165). Kinase-dead (KD) point mutation in the catalytic domain of DNA-PKcs blocks end-ligation without abolishing hairpin opening in knock-in mouse models (166). However, hairpin opening in the DNA-PKcs-KD mice requires ATM kinase activity (166). While pathogenic *PRKDC* mutation in humans is rare, six patients with SCID and DNA-PKcs mutation have been identified, five of whom share mutation of L3062R in the C-terminal FAT domain (85). Interestingly, DNA-PKcs with the L3062R mutation maintains full catalytic activity, but the mutation appears to hinder activation of the Artemis nuclease (167). In addition, one patient with two DNA-PKcs mutations that severely impair (but do not completely ablate) catalytic activity presented both with SCID and a severe neurologic deficit incompatible with life (168). Description of this patient has led to speculation that complete loss of DNA-PK in humans is not compatible with life, and may have a unique function in neuronal development. Deficiencies in Artemis are also associated with SCID with radiation sensitivity (RS-SCID) (169–171).

Once the coding end hairpins are opened, they can be acted upon by nucleases, and extended by error prone polymerases such as V(D)J specific terminal deoxynucleotidyl transferase (TdT) and/or the more general NHEJ polymerases mu and lambda (148, 149, 172, 173). This processing of the coding-end creates additional diversity for antigen selectivity. Finally, the processed coding-ends are ligated by the XLF-XRCC4-LIG4 complex in conjunction with Ku (174). The RSS signal ends are released after the coding-ends and directly ligated by the Ku-XLF-XRCC4-LIG4 complex (175–177). DNA-PKcs, but not Artemis, also plays a role in rejoining of signal ends (166, 178, 179).

Although NHEJ is required for both repairing DSBs produced by IR and those produced by the RAG endonuclease in V(D)J recombination, there are both similarities and differences between the two processes. IR introduces complex forms of DNA damage resulting in DSB ends with diverse sequences and overhanging ends, some of which will contain non-ligatable ends (180). Thus, after IR, NHEJ must be able to 1) respond to DSBs wherever they occur in the genome and 2) hold and tether the ends while they are processed before ligating them. The recently determined structures of NHEJ synaptic complexes reveal how NHEJ proteins can both tether and secure DSB ends while DNA-PKcs autophosphorylation provides a mechanism for handover to end processing enzymes and subsequent ligation by the XLF-XRCC4-LIG4 complex (82, 130, 181–184). In V(D)J recombination, defined DSBs with discrete coding-ends and signal-ends are generated and held within the RAG1/2 heterotetrameric complex (185, 186) before being released and opened by DNA-PKcs-Artemis (coding-ends) and ligated by Ku-XRCC4-LIG4 (coding-ends and signal-ends) (177, 185–187). After hairpin opening, coding ends are processed to include both additional antibody diversity (e.g. TdT) and generate ligatable ends. It will be interesting to determine how the NHEJ machinery interfaces with the RAG1/2 complex and the DNA-PK/Artemis hairpin opening complex.

While the role of Artemis in V(D)J recombination is clear, its role in NHEJ after IR is enigmatic (160). It may act to remove overhanging DNA ends, acting at ds-to-ssDNA transitions as a flap-endonuclease or by direct exonuclease activity and/or it may be required to open secondary structure elements formed by looping of ssDNA at the ends of DSBs. It is likely that Artemis is required for repairing only a subset of DSBs after IR, as Artemis-null cells are not as radiation sensitive as those lacking Ku, XRCC4, LIG4 or DNA-PKcs (148, 188–191).

Animals lacking DNA-PKcs, Artemis, Ku70 or Ku80 are viable but radiosensitive due to defects in NHEJ and immune-deficient due to defects in V(D)J recombination (192–195). For V(D)J recombination in mice lacking functional DNA-PKcs or Artemis, unopened coding-end DNA hairpins accumulate, producing a profound defect in coding joint formation (192, 193, 196). Signal joints are unaffected by loss of Artemis whereas mutation of DNA-PKcs has variable effects on signal joints (157, 175). In SCID horses signal ends are profoundly affected by DNA-PKcs mutation, while SCID dogs and mice have intermediate signal end rejoining, indicating species differences in V(D)J recombination at signal ends, possibly due to relative levels of DNA-PKcs and ATM (165). In contrast, in animals lacking Ku70 or Ku80, both coding and signal joins are affected (194, 195). Mice lacking XRCC4 or LIG4 are non-viable, with embryos undergoing neuronal apoptosis, while cells lacking XRCC4 or LIG4 are radiation sensitive and defective in coding and signal joints, consistent with a more severe V(D)J recombination defect (148, 188–190). Notably, deletion of Ku rescued the embryonic lethality, but not the V(D)J recombination defects in LIG4-null mice, likely through aberrant end-resection and the repair by the Alt-EJ pathway (197, 198).

Besides the DNA-PKcs-Artemis/Ku-XRCC4-LIG4 axis, the MRN-associated kinase ATM plays a critical role in lymphocyte development *via* direct or indirect involvement at various stages of development. Although many details are still unclear, ATM is required for stabilization of the RAG post-cleavage complex that releases the DNA ends to the NHEJ pathway (157, 199, 200). Inactivating somatic *ATM* mutations are associated with T- and B-cell lymphoma (201, 202); dysregulated V(D)J recombination results in translocations in ATM-deficient lymphocytes, potentially promoting tumorigenesis (203, 204). While XLF-deficient cells have significant V(D)J recombination, ATM kinase activity and its chromatin bound DDR factors (e.g., 53BP1 and H2AX), while dispensable for V(D)J recombination in otherwise wild type cells, become essential for chromosomal NHEJ during V(D)J recombination in XLF-deficient cells (205–207). Indirectly, ATM-related repression of GSK3β and cyclin D3 also plays an important role in thymocytes and pre-B cells (208, 209). DSBs generated by both V(D)J recombination and CSR induce ATM-dependent phosphorylation of GSK3β, which is a constitutively active kinase known to promote cell death (209, 210). The inactivation of GSK3β by DSB-initiated Ser^389^ phosphorylation protects B cells during V(D)J recombination and CSR that are required for antigen-specific IgG antibody responses following immunization. During T cell development, GSK3β phosphorylation created by V(D)J recombination also promotes survival of DN3 thymocytes undergoing TCRβ rearrangements, mimicking the results described in mice harboring deficiency in several key DDR factors, including ATM, NBS1 and H2AX (209, 211, 212).

### DDR in CSR and SHM

The DDR is also essential for additional adaptive immune responses that occur after antigen exposure in germinal center B cells. V(D)J recombination-rearranged immunoglobulin (Ig) variable regions are further modified by the process of SHM, after which antibodies with highest affinity are selected. While in CSR, the constant regions of immunoglobulin genes are excised and rearranged to produce other isotypes (e.g. IgA and IgG) from the initially expressed IgM or IgD isotypes (213, 214) (**Figures 2B, C**). Both CSR and SHM are initiated by B cell-specific, activation-induced cytidine deaminase (AID), a member of the apolipoprotein B mRNA editing enzyme catalytic polypeptide like (APOBEC) family of deaminases, which converts cytosine to uracil on single-stranded DNA or RNA (215, 216). Various DDR pathways are then involved in both the generation of strand breaks and their repair.

During SHM, AID deaminates a particular trinucleotide sequence in ssDNA of transcriptionally active genes, leaving behind numerous uracil residues and producing predominantly nucleotide substitutions in rearranged V genes on the heavy- and light-chain loci, and switch (S) regions, which precede most C genes on the heavy chain locus (217, 218). The mutagenic outcome of uracil lesions can then be determined by one of the following DDR responses: (i) Uracil can act as a template for replication, resulting in a fixed C-T transition mutation; (ii) U-G mismatches can be recognized by the error‐prone MMR machinery, in which the MutSα complex (MSH2-MSH6) detects the mismatch and recruits MutLα complex (MLH1-PMS2) to nick the DNA, followed by the recruitment of Polη (DNA polymerase η) to generate mutations (219, 220); (iii) Non‐canonical BER initiated by uracil DNA glycosylase (UNG) can be used to recruit proliferating cell nuclear antigen (PCNA) at the lesions, and low‐fidelity polymerases such as Polη, which can increase mutations during replication of common DNA fragile sites (221), then can be recruited by PCNA ubiquitination and utilized by both MMR and BER resulting in mutagenic repair (149, 222–224). The nick generated by the UNG-dependent BER pathway is particularly important for CSR, as UNG1 knock out largely abolishes CSR (225).

During CSR, DSBs are generated in the switch regions that are subsequently ligated by either the canonical NHEJ pathway or A-EJ pathway which involves XRCC1, MRE11, plus FEN1 (which threads and removes DNA flaps) and Pol theta for which there are inhibitors (114, 149, 226–230). Although many details of this important pathway remain to be determined, it has been suggested that UNG removes AID-incorporated uracil to create an abasic site which is then cleaved by apurinic/apyrimidic endonuclease (APE) to create an SSB. Two closely spaced SSBs on opposite DNA strands can create a DSB (213, 231, 232). Indeed, UNG inhibition sensitizes cells to high APOBEC3B deaminase and to floxuridine (5-FdU), which are toxic to tumor cells through incorporation of 5-FU into DNA (233, 234).

Much of what we have learned about CSR has come from disruption of DNA repair genes in mice leading to immunodeficiency characterized by the production of IgM (the first spliced constant region) but not IgA or IgG (products of CSR) (136). As reviewed recently by Zha and colleagues (191), the most dramatic defects (>90% reduction) in CSR have been observed in mice lacking the tumor suppressor p53-binding protein (53BP1). In contrast, more modest defects (50-70% reduction) occur in animals lacking MDC1, H2AX, Ku, XRCC4, LIG4 and mice lacking DNA-PKcs, Artemis or ATM have only a minor defect (<10%) in CSR (191, 235–239). Yet, a recent report revealed that defects of 3’-flap endonuclease XPF-ERCC1 in B cells impairs A-EJ-mediated CSR by impeding joining of resected 3′ flap DSB ends (240). Since 53BP1 and Shieldin both block resection and promote NHEJ, loss of either would be expected to promote resection over NHEJ (241–245). However, even loss of LIG4, which abolishes NHEJ, decreases CSR by only 70% (236, 238). A major feature of CSR is removal of large regions of chromatin between the switch regions to be joined. 53BP1 plays a role in looping DNA at telomeres and is required for rejoining of distal joins in V(D)J recombination (246–248), suggesting that long-range conformational changes in DNA may be disrupted in 53BP1-deficient cells, possibly explaining the importance of 53BP1 in CSR (249, 250). Indeed, loss of components of the shieldin complex, which protects DSB ends to mediate 53BP1-dependent repair, also yield defects in CSR (242, 245, 250–252). Alternatively, 53BP1 also recruits PTIP, an evolutionarily conserved chromatin regulator that binds γH2AX, acts as a major effector of ATM and ATR signaling mechanisms, and is also implicated in CSR (253, 254).

Besides the direct usage of DDR pathways, there are several indirect links between DDR elements in CSR and SHM. For instance, targeting HR by RAD51 inhibitor reduces AID expression, hampering the repair of AID-initiated lesions (255). Interestingly, indirect links reach into RNA-binding proteins such as the autism-associated protein vigilin, which interacts with RAD51 and BRCA1, so its depletion impairs their recruitment to DSB sites (256).

## DDR Inhibition in Antitumor Immunity

**Figure 3:**
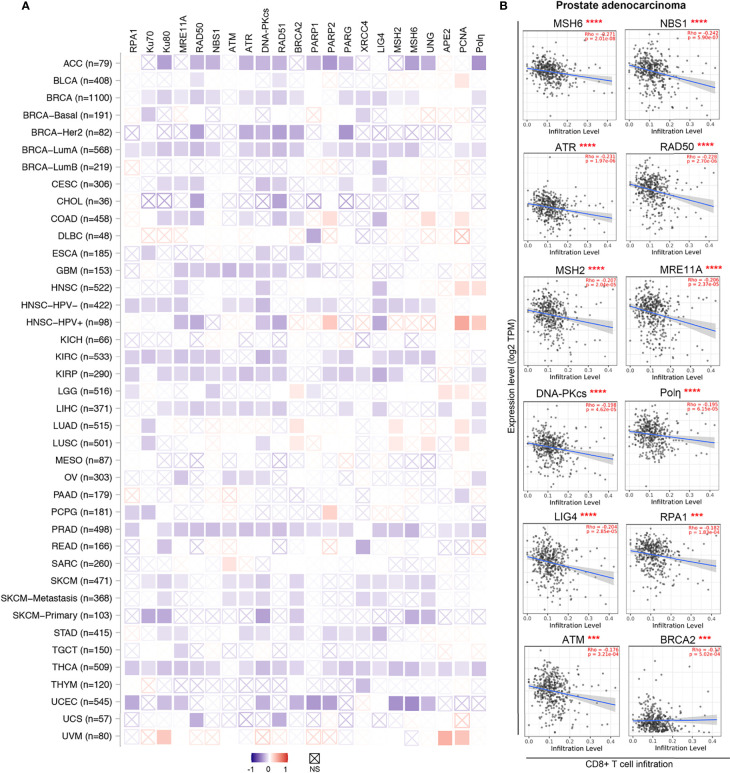
DDR factors negatively associate with CD8+ T cells infiltration levels in diverse cancer types. **(A)** A heatmap based on the CIBRSORT method shows the purity-adjusted Spearman’s rho of DDR factors with CD8+ T cells across various cancer types. The boxes with indicate non-significant p values (p>0.05). The figures was made using the TIMER2.0 web server based on CIBRSORT analysis (http://timer.cistrome.org/). **(B)** Detailed correlation between DDR factors and CD8+ T cells in prostate adenocarcinoma (PRAD) from panel **(A)** The purity-adjusted Spearman’s rho and p value are labeled in red. ***p < 0.001; ****p < 0.00001.

Emerging evidence supports the idea that DDR inhibition in tumor cells remodels the inflammatory microenvironment (10, 257). Impaired DDR typically enhances the tumor foreignness by increasing the number of tumor cell mutations/neoantigens (10, 258). When examined by CIBERSORT analysis through the TIMER2.0 web server (259, 260), the mRNA levels of many DDR factors, such as *RPA1*, *Ku70*, *Ku80*, *MRE11A*, *RAD50*, *NBS1*, *PRKDC*, *RAD51*, *PARG* and *XRCC4*, were negatively associated with cytotoxic CD8+ T cells infiltration levels across various cancer types (**Figure 3A**). Indeed, as exemplified in prostate adenocarcinoma, significant negative correlations between gene expression and cytotoxic T cell infiltration levels were found in 19 genes of the 22 DDR related genes we tested (**Figure 3A, B**). These findings suggest enhanced anticancer immunity in tumors with lower DDR factor expression and imply substantial potential benefits from DNA repair inhibitors. Thus, inhibitors of these DDR factors, such as poly(ADP-ribose) glycohydrolase (PARG) inhibitors that impact DNA break repair and replication fork restart, may be employed to activate the innate immune response (261).

**Figure 4:**
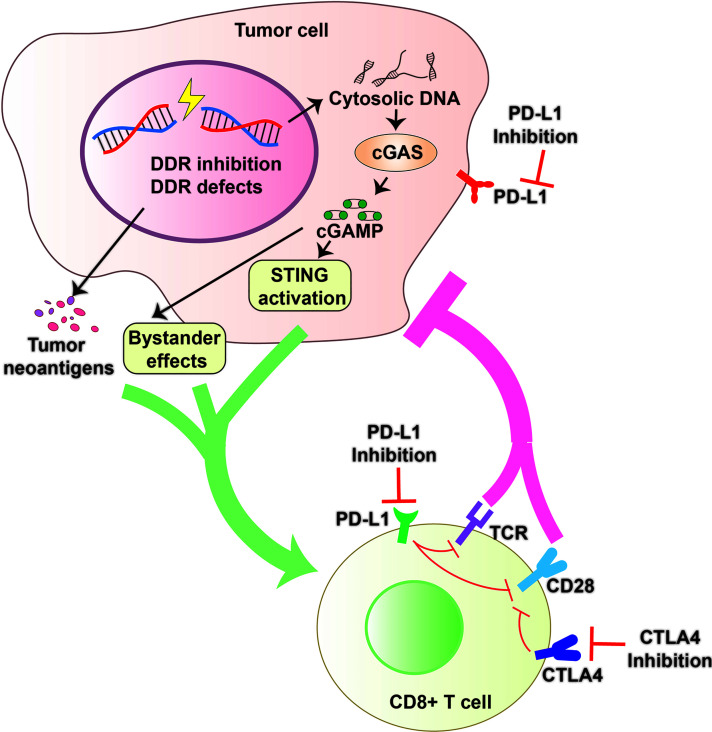
DDR Inhibition and Antitumor Immunity. DDR Inhibition and DDR defects can increase cytosolic DNA that activates the cGAS to generate cGAMP and promote tumor neoantigen production. cGAMP can activate cell intrinsic STING pathway and spread the immunity to bystander cells. All these factors contribute to an inflammatory tumor microenvironment and promote the recruitment of cytotoxic CD8+ T cells and constrict cancer growth effectively. Combining DDR inhibition (such as PARP or PARG inhibition) with Immune checkpoint blockade (including PD-1/PD-L1 or CTLA4 blockade) may be a promising strategy with the potential to improve survival outcomes.

Antitumor immune responses can be promoted and utilized to treat cancer *via* immune checkpoint blockade with use of agents such as PD-1/PD-L1 and CTLA-4 inhibitors (29, 262, 263). The DDR also offers attractive targets for inhibition (264, 265). Preclinical and clinical efficacy of DDR inhibition in cells with a defective DDR genetic background, are exemplified by the success of PARP inhibitors in *BRCA1/2*-mutated advanced cancers and of inhibitors to the PARG in cancer cells (261, 266, 267). Emerging evidence has progressively unveiled the involvement of the DDR in antitumor immunity by enhancing STING-dependent immune responses, further supporting the immune-modulatory role of DDR inhibition in anticancer immunity (**Figure 4**) (134, 135, 268, 269).

The most studied DDR inhibitors in anticancer immunotherapies are those directed against PARP (PARPi). In line with the usage of PARPi in DDR-deficient tumors (266), PARPi combined with immune checkpoint blockade, including PD-1/PD-L1 and CTLA-4, exerts remarkable efficacy in tumors with BRCA1/2 or ERCC1 mutations *via* STING-dependent immune responses and infiltration of cytotoxic T cells into tumor (50, 51, 54, 270). There are also findings suggesting that PARPi, with anti-PD-1 inhibitors, have strong therapeutic potential regardless of BRCA1/2 status (49, 271, 272), although the mechanisms involved remain unclear. Besides the STING-dependent pathways, PARPi also increased PD-L1 expression in breast cancer cell lines through inhibition of GSK3β (273), which provided the rationale for combining PARPi with PD-L1 or PD-1 immune checkpoint blockade, a strategy that has been tested in clinical trials (49, 271, 274).

Recently, many other inhibitors targeting DDR components have been developed and are in preclinical study. Recently, several of them, including inhibitors of DNA-PKcs, ATM, ATR, CHK1 and WEE1, have entered into clinical trials (275). Inhibitors of DNA-PKcs promote radiation sensitization through inhibition of NHEJ (276). Their importance in modulating the innate immune response have also been demonstrated. ATR inhibition can further increase cGAS-positive micronuclei and cytokine production in PARPi-treated cancer cells (12). Significantly, inhibition of DNA-PK with AZD7648 resulted in IFN-dependent inhibition of tumor growth following IR in immune competent mouse models, indicating that inhibition of DNA-PK in combination with radiotherapy could lead to durable immune-mediated tumor control in cancer patients (277).

Another important application of DDR in antitumor immunotherapy is the usage of the DDR status as biomarkers to select the patients who are targetable to immune checkpoint blockade. Currently, only a subset of patients respond to immune checkpoint blockade. Predictive biomarkers for reliable response could better guide therapeutic choices (104). As DNA repair deficiencies that promote genome instability are relatively common among tumors, mutational signatures and DDR biomarkers may identify features associated with response to immune-directed therapies. For instance, MMR status was reported to predict response to the PD-1 inhibitor pembrolizumab in a phase 2 study of 41 patients with progressive metastatic carcinoma (278, 279). Also, loss of BRCA1 and defects of MMR in tumors resulted in many somatic mutations, leading to continuous renewal of neoantigens, increased immune response gene expression, and enhancement of immune surveillance (20, 270, 278, 280). In non–small cell lung cancer, deleterious mutations in several DDR-related genes correlated with pembrolizumab clinical efficacy (281). A high mutation level causing a high load of tumor neoantigens suppresses immune evasion. Whereas aneuploidy of large chromosomal regions (arm and whole-chromosome), which cause somatic copy number alterations (SCNAs) and consequent protein imbalances, can weaken cytotoxic immune cell infiltration (282). Importantly, blockade of the immune system PD-1/PD-L1 inhibitory pathway can restore exhausted immune responses as an effective immunological strategy to overcome immune evasion by chronic imbalances and infections (283). For monoclonal antibodies used to block checkpoint molecules, such as PD-1 and PD-L1, to activate immune cells to kill tumor cells more effectively, it may be worth adding designed features such as metal ion binding sites to add to their capabilities or removing free cysteines to improve their stability (284–286).

## Summary and Prospects

The DDR shapes how the innate immune system responds to tumors, as well as how the adaptive immune system is recruited to sites of malignancy. Consequently, the interconnections of the DDR and the immune system, which maintain genomic fitness and pathogen protection, can be utilized to improve cancer therapeutic strategies (5, 135, 287–291). Yet, defining how the DDR impacts immune responses has remained challenging as immune activation can evidently be triggered by different types of DDR components including DNA damage sensors, transducers, and effectors (292).

Here, we assessed current molecular and mechanistic data showing how the DDR induces and impacts immune responses. At present, cancer immunotherapy is less widely used than surgery, chemotherapy, or radiation therapy. As only a subset of patients respond to immune checkpoint blockade, enhancements from defining and modulating the DDR along with reliable predictive biomarkers of response are needed to guide and improve therapeutic strategies. DNA repair deficiency is common among tumors, and emerging experimental and clinical evidence suggests that features of genomic instability are associated with response to immune-directed therapies. We propose that advancing all successful cancer therapies will benefit from elucidating key molecular and mechanistic relationships linking DDR, DNA damage outcomes, and immune responses. In fact, the efficacy of conventional chemotherapy and radiotherapy can depend in part upon induction of innate and adaptive immunity.

In innate immunity (**Figure 1**), MRN (along with its associated ATM and ATR kinases) and DNA-PK complex, which co-regulates DNA DSB repair, can serve as master cytosolic DNA sensors to initiate innate immune response. DNA-PKcs expression with validated immune biomarkers can guide patient selection for DNA-PKcs targeting strategies, DNA-damaging agents, and their combination with an immune-checkpoint blockade (293). Analogously, ATM inhibition induces tumor growth delay and overcomes tumor resistance to anti–PD-1 therapy (294). In addition, other DDR components interact with and promote cytosolic DNA sensing pathways or RIG-I–mediated RNA sensing signaling to trigger innate immune response. Whereas mice and other model systems have proven to be of great value for testing these molecular mechanisms, it is critical to consider possible impacts from the far higher DNA-PKcs levels in human cells compared to laboratory mice (104).

Most immune-related DDR components and immune responses converge upon the STING-IFN signaling pathway, which plays a crucial role in cancer cell immune-surveillance. In adaptive immunity (**Figure 2**), DDR pathways (including MMR, BER, NHEJ, and A-EJ) are required for V(D)J recombination, CSR and SHM processes, which are critical to lymphocyte development. From a pathology standpoint, DDR modulates anticancer immunity *via* both innate and adaptive immunity, with the underlying molecular mechanisms being increasingly defined. Such knowledge is likely broadly applicable to human disease, including cancer, infectious disease and atherosclerotic disorders. For instance, SARS CoV-2 proteins, can hijack the human immune response to pathogens and the DNA damage repair system, thereby damaging both innate and adaptive immunity (295, 296). Furthermore, the results of targeting endonuclease V, a ROS response and structure-specific nuclease that cleaves DNA and RNA at inosines as a regulator of innate immune responses, suggests blocking such DDR-related epitranscriptomic modifications to ameliorate carotid atherosclerosis and ischemic stroke (297–299).

For advanced immunotherapeutic strategies, DDR defects plus the increased mutation load in tumor cells produce tumor-specific neoantigens. So chemical tools to alter the DDR in predetermined ways can leverage the full power of cancer immunotherapy. Importantly, advances in structural biology for combining atomic resolution structures with X-ray scattering and computation for solution conformations and assemblies are providing critical enabling methods to define and target dynamic complexes that can generally control mutation rates (66, 300–302). We propose here that the dynamic DNA-PK and MRN-activated ATM and ATR are potential master keys to unlock DDR and their immune system roles. As DNA-PKcs, ATM, and ATR inhibitors are already being evaluated in clinical trials as sensitizers of chemotherapy and radiotherapy, we suggest that these kinases may be both a predictive biomarkers and therapeutic targets for immunotherapy in future clinical trials.

To effectively use such master keys, it will be important to better define the molecular mechanisms orchestrating their activities in DDR and immune system outcomes and their potential as biomarkers for prognosis. We know that with molecular mechanistic knowledge, examination of DDR status can provide informed predictive biomarkers for patient selection and therapeutic approaches (135). Moreover, like immune checkpoint inhibitors, DDR inhibition strategies show great potential to improve cancer treatment efficacy by harnessing their immunomodulatory effects for radiation and chemotherapies, immune checkpoint blockade, and combined therapeutic strategies.

## Author Contributions

ZY and YS contributed equally to write the original draft. JT and SL-M contributed equally to the conceptualization and revising. All authors listed have made direct and substantial contribution to this work, and approved the final manuscript.

## Funding

Our research was supported by National Institutes of Health (NIH) grants (R01 CA200231; P01 CA092584; R35 CA220430; 1S10OD012304-01) and by Cancer Prevention Research Institute of Texas (CPRIT) grant (RP180813). JT’s efforts are also supported by a Robert A. Welch Chemistry Chair. YS’ efforts are supported by research grants from National Natural Science Foundation of China (Grant No. 31801161).

## Conflict of Interest

The authors declare that the research was conducted in the absence of any commercial or financial relationships that could be construed as a potential conflict of interest.

## Publisher’s Note

All claims expressed in this article are solely those of the authors and do not necessarily represent those of their affiliated organizations, or those of the publisher, the editors and the reviewers. Any product that may be evaluated in this article, or claim that may be made by its manufacturer, is not guaranteed or endorsed by the publisher.

## Glossary

5-FdU: floxuridine
53BP1: p53-binding protein
8-OHG: 8-hydroxyguanosine
A-EJ: alternative end joining
AgR: antigen receptor
AID: activation-induced cytidine deaminase
APE2: Apurinic/apyrimidinic endodeoxyribonuclease 2
APOBEC: apolipoprotein B mRNA editing enzyme catalytic polypeptide like
ATM: ataxia telangiectasia mutated
A-T: Ataxia-telangiectasia
BCL10: B cell CLL/lymphoma 10
BER: base excision repair
BLM: bloom syndrome RecQ like helicase
BRCA1/2: breast-cancer susceptibility gene 1/2
BS: bloom syndrome
CARD9: caspase-recruitment domain
CHK1: checkpoint kinase 1
CTLA-4: cytotoxic T-lymphocyte-associated antigen 4
cGAMP: 2’-3’cGAMP
cGAS: cyclic GMP-AMP synthase
CSR: class-switch recombination
DAMPs: damage-associated molecular patterns
DDR: DNA damage response
DNA-PK: DNA-dependent protein kinase
DSBs: double-strand breaks
EEPD1: endonuclease/exonuclease/phosphatase family domain containing 1
dsDNA: double-stranded DNA
ERCC1: excision repair cross complementary gene 1
EXO1: exonuclease 1
EXO5: exonuclease 5
FANCD2: Fanconi anemia complementation group D2
FEN1: flap structure-specific endonuclease 1
GSK3β: glycogen synthase kinase 3 beta
GRB2: growth factor receptor bound protein 2
H2AX: H2A histone family member X
HDP-RNP: HEXIM1-DNA-PK-paraspeckle components-ribonucleoprotein complex
HIV: human immunodeficiency virus
HMGB1: high mobility group box 1
HR: homologous recombination
HSPA8: heat shock protein family A (Hsp70) member 8
HSV-1: herpes simplex virus 1
Ig: immunoglobulin
IRF3: interferon regulatory factor 3
IFI16: IFN-inducible protein 16
IFN: interferon
LAT: Linker of Activation of T cells
LIG4: DNA ligase IV
LINP1: lncRNA in nonhomologous end joining (NHEJ) pathway 1
IL7: interleukin 7
IR: ionizing radiation
MAVS: mitochondrial antiviral signaling protein
MDC1: Mediator of DNA damage checkpoint 1
MMR: mismatch repair
MLH1: MutL homolog 1
MRE11: meiotic recombination 11 homolog 1
MRN: MRE11-RAD50-NBS1
MSH2: MutS homolog 2
MSH6: MutS homolog 6
MUTY: MutY DNA glycosylase
NBS1: Nijmegen breakage syndrome protein 1
NEIL: endonuclease VIII (Nei)-like proteins
NER: nucleotide excision repair
NF-κB: nuclear factor kappa B subunit 1
NHEJ: non-homologous end joining
OGG1: oxoguanine DNA glycosylase
p53: tumor protein p53
PARG: poly(ADP-ribose) glycohydrolase
PARP: poly (ADP-ribose) polymerase
PARPi: PARP inhibitors
PAMPs: pathogen associated molecular patterns
PCNA: proliferating cell nuclear antigen
PD-1/PD-L1: programmed cell death protein 1/programmed cell death ligand 1
PIKK: phosphatidylinositol 3-kinase (PI3K)-related kinase
PMS2: PMS1 homolog 2
Polη: DNA polymerase η
pre-BCR: pre-B cell receptor
PRRs: pattern recognition receptors
PRKDC: Protein kinase, DNA-activated, catalytic polypeptide
RAD50: ATP-binding cassette (ABC)-ATPase 50
RAD51: ATP-binding cassette (ABC)-ATPase 51
RAG: recombinase activating gene
RIG-I: retinoic acid-inducible gene I
RPA: replication protein A
ROS: reactive oxygen species
RSS: recombination signal sequences
SARS CoV-2: severe acute respiratory syndrome coronavirus-2
SCID: severe combined immunodeficiency
SHM: somatic hypermutation
SCNAs: somatic copy number alterations
SIDSP: STING-independent DNA sensing pathway
SSBs: single-strand breaks
STING: stimulator of interferon genes
TBK1: TANK-binding kinase 1
TCRβ: T cell receptor beta
TdT: terminal deoxynucleotidyl transferase
TREX1: three prime repair exonuclease 1
WEE1: WEE1 G2 checkpoint kinase
XLF: XRCC4-like factor
XRCC1: x-ray repair cross-complementing group 1
XRCC4: x-ray repair cross-complementing group 4
UNG: uracil DNA glycosylase
V(D)J: variable, diversity, and joining.
